# First person – Hao Lu

**DOI:** 10.1242/bio.052951

**Published:** 2020-05-18

## Abstract

First Person is a series of interviews with the first authors of a selection of papers published in Biology Open, helping early-career researchers promote themselves alongside their papers. Hao Lu is first author on ‘ Reissner fiber-induced Urotensin signaling from CSF-contacting neurons prevents scoliosis of the vertebrate spine’, published in BiO. He is a Research Fellow in the laboratory of Dr Sudipto Roy at the Institute of Molecular and Cell Biology in Singapore, using the zebrafish and mouse as animal models to study ciliogenesis and ciliopathies.


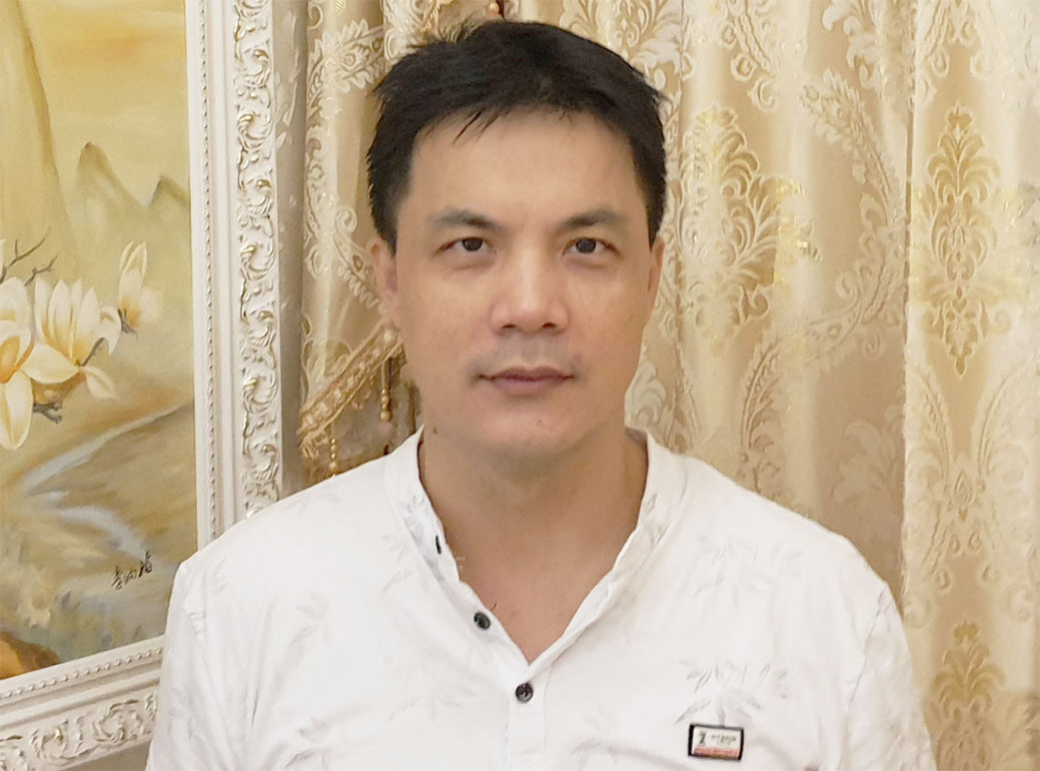


**Hao Lu**

**What is your scientific background and the general focus of your lab?**

I have a research background related to metabolism, developmental biology, ciliogenesis and ciliopathies. During my PhD studies, I studied mitochondrial metabolism, and identified a small protein, GRIM19, as a novel component of mitochondrial complex I. After I graduated, I joined Dr Sudipto Roy's lab because I realized that the zebrafish is an ideal genetic system to study vertebrate development and model human disease. The Roy lab focuses on understanding many aspects of developmental biology and human disease, especially ciliogenesis and ciliopathies. For example, our group has uncovered the master regulatory role of the FoxJ1 transcription factor in motile cilium generation, and we established the Gmnc and Mcidas transcription factors as key regulators in programming the differentiation of multiciliated cells. Currently, our lab uses the zebrafish and the mouse to study the mechanistic basis of human ciliary diseases, such as left–right asymmetry, polycystic kidney disease and idiopathic scoliosis (IS).

**How would you explain the main findings of your paper to non-scientific family and friends? **

IS is a common human spine disorder, affecting up to 3% of children and adolescents worldwide. It manifests in three-dimensional curvatures of the spine, causing disfigurement of the torso and chronic back pain as well as postural and gait problems. Despite being a highly prevalent and debilitating disorder, we do not fully understand the pathological basis of the disease. Current treatment options are largely limited to wearing corrective braces or invasive surgery, particularly in cases with acute deformations. Using genetic analysis in the model organism zebrafish, we show that a thread-like structure called Reissner fiber (RF), which remains suspended in the cavities of the brain and spinal cord and bathed in cerebrospinal fluid (CSF), is required to induce the expression of the Urotensin family neuropeptides in a special set of nerve cells that are also in contact with CSF, the CSF-contacting neurons (CSF-cNs). Earlier work from our group and those of others had shown that tiny hair-like projections called cilia, which continuously beat within the brain cavities and spinal canal, helps to transport CSF that contains hormones like epinephrine. RF is able to bind epinephrine, and we think it is through this mechanism that it stimulates Urotensin neuropeptides in CSF-cNs. The CSF-cNs then use these neuropeptides to signal to special kinds of muscle cells of the trunk and tail, and contractile activity of these muscles fibers is the likely cue that directs proper morphogenesis of the spine. We believe that these findings will enable us to better understand how IS develops, and importantly, novel treatment options could become available through pharmacological exploitation of this knowledge.

“…we show that a thread-like structure called Reissner fiber (RF)… is required to induce the expression of the Urotensin family neuropeptides in a special set of nerve cells that are also in contact with CSF…”

**What are the potential implications of these results for your field of research?**

The etiology of IS currently remains elusive (hence the term idiopathic, meaning without a known cause). Our earlier research with the zebrafish and those from other labs had implicated several factors such as cilia, RF and Urotensin signaling in regulation of spine morphogenesis. Our current work bridges existing gaps in our knowledge between these factors and spine deformities. It will be important to investigate to what extent these factors are the causal basis of IS. If conserved, we can consider that modulation of epinephrine activity as well as targeting the Urotensin pathway could provide novel therapeutic avenues for the disorder.

**What, in your opinion, are some of the greatest achievements in your field and how has this influenced your research?**

Even though cilia were known from the 17th century – the time of Antonie van Leeuwenhoek, the father of the microscope – I think one of the greatest achievements in the cilia field has been the realization, over the past two and half decades or so, that these tiny hair-like organelles are important for a range of biological functions. This has largely stemmed from observations that ciliary dysfunction is directly linked to various human diseases, like polycystic kidney disease, airway disease, congenital heart disease and many others, collectively termed ciliopathies. Interestingly, like cilia, RF was also discovered more than a century ago by the German anatomist Ernst Reissner, but its function had remained enigmatic. Our work and that of other labs is now illuminating, for the first time, the importance of RF in signaling functions within the nervous system that is critical for proper spine morphogenesis. Thus, it is fascinating to reflect on the similar histories of the cilium and RF, largely mired in obscurity, and the present unification of their functions in the regulation of spine morphogenesis. I feel extremely privileged in being able to contribute to this relatively new area of biology. If, at some point in the future, any of my research findings with cilia and RF go on to make a difference in the lives of patients with IS, that would indeed be most gratifying.

**Figure:**
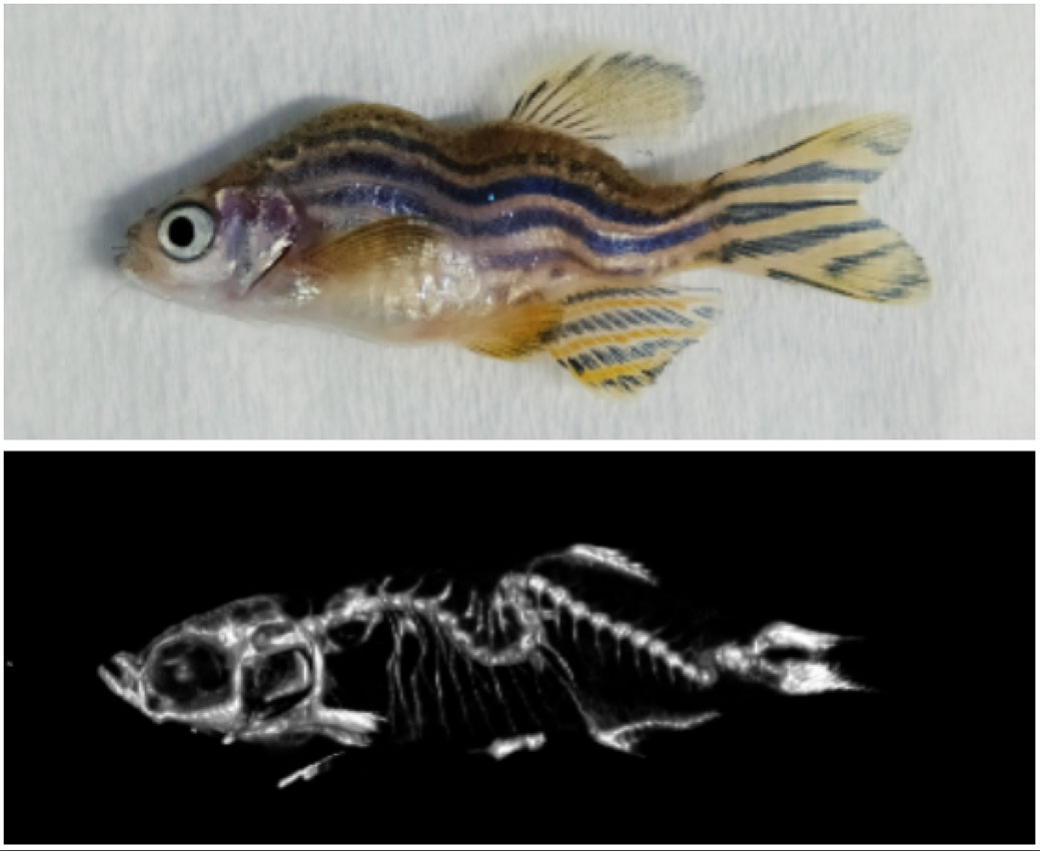
**Zebrafish mutants lacking Reissner fiber (RF) develop into adults with scoliotic spines, resembling the common human spine disorder idiopathic scoliosis.** RF mutant zebrafish (top panel); microCT scan of the spine (bottom panel). Note the spinal curvatures.

“RF was also discovered more than a century ago by the German anatomist Ernst Reissner, but its function had remained enigmatic.”

**What changes do you think could improve the professional lives of early-career scientists?**

Currently, funding for research, especially for basic research, is quite limited and is dwindling by the day. As an early-career scientist, it is very important to work smart and work hard due to intense competition from very talented minds. Working smart includes the need to read the appropriate literature, network with other researchers and regularly attend different seminars and conferences to gain first-hand knowledge of the latest developments in our fields of interest. All of these activities will enable us to stay focused on important questions and gain sufficient support and mentoring from experts. Given the issue of limited resources available to early career scientists worldwide, I feel opening up new avenues of research funding to specifically cater to this group of people and a healthy balance of fund allocation between basic and translational research will go a long way to mitigate some of the constraints that we are currently facing.

**What's next for you?**

As a first step towards becoming an independent researcher, I am currently applying for a few competitive grants. I want to continue to investigate the relevance of the zebrafish data on spine deformations to the etiology of IS. In addition, I have some interesting data with regard to polycystic kidney disease and cilia, which might also be useful for translational research. I am also striving hard to find a balance between basic and applied research, as we are under increasing pressure from government agencies that fund our work to make our investigations more aligned with biomedical relevance. As a scientist, I am motivated to pursue the discovery of new knowledge and I remain excited at the potential of practical applications of my research findings.
